# Staff Assessment Person-Directed Care Questionnaire: Adaptation and Validation for the Portuguese Population

**DOI:** 10.1177/23337214221103394

**Published:** 2022-06-04

**Authors:** Maria M. Barbosa, Laetitia Teixeira, Javier Yanguas, Constança Paul, Rosa M. Afonso

**Affiliations:** 1 Instituto de Ciências Biomédicas Abel Salazar, School of Medicine and Biomedical Sciences, University of Porto, Rua Jorge de Viterbo Ferreira, 228, 4050-313 Porto, Portugal; 2 Health Sciences Research Centre, Faculty of Health Sciences, University of Beira Interior (CICS-UBI), Avenida Infante D. Henrique, 6201-506 Covilhã, Portugal; 3 Center for Health Technology and Services Research (CINTESIS), Rua Dr. Plácido da Costa, s/n, 4200-450 Porto, Portugal; 4 Aubixa Fundazioa, 20009 Donostia-San Sebastian, Spain; 5 Department of Psychology and Education, Faculty of Human and Social Sciences, University of Beira Interior, Estrada do Sineiro, s/n, 6200-209 Covilhã, Portugal

**Keywords:** person-centered care, residential care facilities, geriatrics, aged, questionnaire validation

## Abstract

Person-centered care aims to increase and guarantee the quality of care at residential care facilities for older adults. The implementation and development of this approach requires validated assessment tools, which are still lacking in Portugal. This study aims to adapt and validate for the Portuguese population the internationally and widely used essential instrument that is the Staff Assessment Person-Directed Care (SAPDC). The adaptation of the SAPDC included its translation, back translation, and a pilot-study. For validation, staff members were recruited by distributing the study via email and on social media. Respondents included 546 native Portuguese-speaking staff members working at residential care facilities for over 6 months. The mean score of SAPDC was 165.74 (SD = 36.78). The exploratory factor analysis showed eight conceptually distinct dimensions, considered adequate by the expert team. The total scale showed a very good internal consistency (α = .96) and excellent temporal stability assessed by Intraclass Correlation Coefficient (> .90). Providing a Portuguese version of the SAPDC is useful to substantiate technical and scientific advancements and define policies with implications on evolving care approaches. This tool helps optimize the quality and dignification of gerontological practices, which is urgent at Portuguese residential care facilities.

## Introduction

Residential care facilities (RCFs) for older adults have evolved throughout the years. Gerontology-related literature, however, emphasizes a growing concern over the type of care developed at RCFs, which usually focuses on procedures and standards that jeopardize the humanization of practices (Caspar et al., 2020; Fernández-Ballesteros et al., 2019; Lood et al., 2020).

This is frequently called “traditional care” (Li & Porock, 2014) and focuses mainly on task and routine management at RCFs. The needs of facilities are prioritized over the needs, idiosyncrasies and preferences of residents. Traditional care is usually guided by sanitary criteria, where age and dependence are homogenizing factors leading to an uniformization of practices (Barbosa et al., 2021; Caspar et al., 2020; Sánchez-Izquierdo et al., 2019). This approach may lead to paternalistic attitudes, meaning that older adults are seen as passive recipients of care, and caretakers assume a dominant attitude with protective intentions, making decisions for the care receivers (Fernández-Ballesteros et al., 2019). Traditional care usually leads to the disempowerment of older adults and to violations of their rights, worsening the negative impacts on their well-being (Associação Portuguesa de Apoio à Vítima, 2020; Fernández-Ballesteros et al., 2019; Gil, 2019). This care shows signs of low quality and low sustainability and has been criticized and rejected (Martínez et al., 2019; Martínez, Suárez-Álvarez, Yanguas, et al., 2016). Therefore, awareness over the need to evolve care approaches is growing. Person-centered care has been identified as an alternative to traditional care (Barbosa et al., 2021; Lood et al., 2020; Martínez et al., 2019).

Person-centered care is rooted in humanism. In the 1980s Tom Kitwood promoted the use of this approach in care provided to people with dementia (Caspar et al., 2020; Fernández-Ballesteros et al., 2019). Person-centered care later became an international reference and is now recommended as a global strategy to drive change in care culture at RCFs, representing the highest standard of care to older adults, regardless of frailty, pathologies or dependency level (Caspar et al., 2020; Edvardsson et al., 2017; Sköldunger et al., 2020; Yevchak et al., 2019).

Person-centered care emphasizes the value of each individual as a singular human being, who should be placed at the center of the care dynamic (Caspar et al., 2020; Díaz-Veiga et al., 2016; Sköldunger et al., 2020). Meaning, care should be personalized to each individual’s needs, preferences and biography (Caspar et al., 2020; Martínez, Suárez-Álvarez, Yanguas, et al., 2016; Sköldunger et al., 2020). Care is developed cooperatively and the person is perceived as an active and integral agent in the process of care (Fernández-Ballesteros et al., 2019; White et al., 2008). Person-centered care highlights the importance of promoting autonomy by providing opportunities for making decisions and taking risks (Caspar et al., 2020; Lood et al., 2020; Sköldunger et al., 2020). Literature also mentions person-centered care elements related to significant relationships with staff (Lood et al., 2020), physical environment (Edvardsson et al., 2010; Lood et al., 2020; White et al., 2008) and various organizational variables (Edvardsson & Innes, 2010; Hunter et al., 2015). Person-centered care promotes the rights of residents (Barbosa et al., 2021), has a positive impact on their well-being and quality of life, and reduces staff member strain (Caspar et al., 2020; Sköldunger et al., 2020; Sullivan et al., 2012).

Implementation and monitoring person-centered care requires validated measurement tools which are adapted to the cultural settings (Fernández-Ballesteros et al., 2019; Kazemi & Kajonius, 2021; Martínez, Suárez-Álvarez, & Yanguas, 2016). From the existing tools, the questionnaires used to obtain the opinions of staff must be highlighted, as staff is responsible for the care practices and the changes required to apply person-centered care (Edvardsson et al., 2010; Martínez et al., 2015; White et al., 2008). The Staff Assessment Person-Directed Care (SAPDC) is one of the most relevant instruments used in related international studies (White et al., 2008), and has shown appropriate psychometric properties in different studies (e.g., Martínez, Suárez-Álvarez, Yanguas, et al., 2016; Sullivan et al., 2012; White et al., 2008).

There’s a gap in the field of person-centered care measurement instruments in Portugal, where the SAPDC has not yet been validated. This study seeks to adapt and validate the SAPDC for the Portuguese population, which will be critical to advance research and practice.

## Methods

### Ethics

This study integrates the project “Atenção Centrada na pessoa na prestação de cuidados na velhice: abordagens e instrumentos de avaliação” and was approved by the Ethics Committee from Universidade da Beira Interior (n° CE-UBI-Pj-2019-057-ID1555). The validation protocol included an informed consent containing the context and objectives of the study, a guarantee of confidentiality, voluntariness, and the availability of a contact person within the investigation team for clarification. Anonymity and confidentiality were assured in the data collection.

## Materials

The SAPDC is a person-centered care measurement instrument originally developed for the American population by White et al. (2008). While developing the instrument’s items, the authors revised the existing literature and identified two large clusters: person-centered care central components and physical/organizational environment, an essential component for supporting practices (White et al., 2008). After establishing the items according to these topics, they performed two sets of data analyses, each applied to a different cluster. These analyses identified eight factors: five related to person-centered care (Autonomy, Personhood, Knowing the Person, Comfort Care, Supporting Relationships) and three related to the physical/organizational environment (Work with Residents, Personal Environment for Residents, and Management Structure).

The final version of the SAPDC has 50 items and a 5-point likert-type answer scale ranging from “very few” or “none/rarely” or “none of the time” to “all or almost all/all” or “almost all of the time” (White et al., 2008). The SAPDC is answered individually, easily applicable and completion is estimated for under 15 minutes (Sullivan et al., 2012). The instrument is oriented to staff members working directly and indirectly with residents. Where no work is done directly with residents (e.g., administration, maintenance), participants are instructed to provide their opinion about how the RCF is run. This instrument provides a general score and independent scores for each factor. The higher the score, the higher the degree of person-centered care practices applied at RCFs according to staff (Sullivan et al., 2012).

### Target Population

Considering the SAPDC’s purposes and indications, this study targets the staff members of Portuguese RCFs. In Portugal, there are about 2500 RCFs integrated in the network of social services and facilities, with over 100,000 residents (Ministry of Labor, Solidarity and Social Security, 2020). About 80% of RCFs are Private Institutions of Social Solidarity (non-profit organizations formed exclusively through the initiative of entities and supported by the social security system), and about 20% are for profit. Although no official numbers exist, over 60.000 staff members are estimated to work at Portuguese RCFs (National Health Service, 2020).

RCFs in Portugal need a license from the Social Security Institute and can be described as collective housing structures for people aged 65 or over. They provide services related to social support, meals, hygiene, health care and support in performing daily activities. These structures are managed by technical directors in charge of programming institutional dynamics and supervising staff members, like nurses, entertainment coordinators, psychologists and direct-care workers (Ministry of Labor, Solidarity and Social Security, 2012).

### Procedures

After receiving the authors’ authorization for adapting and validating the SAPDC for the Portuguese population, a guide was created using the guidelines and good practices of Borsa et al. (2012), Cardoso (2006), International Test Commission (2017) and Sousa and Rojjanasrirat (2011). This set resulted in the development of two stages and 11 procedures (Table 1).

**Table 1.** Stages and Procedures for Staff Assessment Person-Directed Care Adaptation and Validation.

Stage/Procedure Adaptation	Description
1. Translation	Two independent translators translated the SAPDC into Portuguese
2. Translation comparison	The two versions were compared and summarized and their discrepancies were resolved with the translators
3. Preliminary version analysis	Four experts analyzed the layout, any sources of conflict and equivalences of the preliminary version, which was later analyzed by a Portuguese language expert
4. Preliminary version testing	At individual interviews, 11 participants were asked to assess the instrument out loud and present suggestions until saturation of data
5. Instrument’s back translation	Two independent translators blindly translated the instrument from Portuguese into English and the discrepancies were resolved with them
6. Back translation analysis	The original authors analyzed and validated the Portuguese version of the SAPDC.
7. Pilot-study	19 staff members classified the instrument’s components as “clear/unclear”. An agreement of 80% was achieved between evaluators on all fractions (criteria considered by Sousa & Rojjanasrirat, 2011 for fraction maintenance)
Validation	
8. Contact base development	The most official resource that unifies information on social responses (the social charter for continental Portugal and the webpages of social security institutes from the islands) was used and the available RCFs’ emails were collected
9. Participant recruitment	To disseminate the study, 2325 emails were sent and social networks were used. The information contained a presentation of the project, a link to the questionnaire and a request for sharing among staff at RCFs with the following inclusion criteria: Working at RCFs for more than 6 months, having Portuguese as native language and accepting the commitment to participate. To include low digital literacy staff, RCFs had the opportunity to request printed questionnaires
10. Data collection	Data collection took place from April to July 2021, when the sample was sufficient to comply with the International Test Commission (2017) recommendation of at least 10 participants per item. 17 facilities required paper questionnaires, 305 were sent by post, and 211 were returned (100 were considered valid)
11.Test-retest	The instrument was reapplied 7 days later to a convenience sample of 25 staff members. Anonymity was safeguarded through a double-encrypted personal code

SAPDC: Staff Assessment Person-Directed Care; RCF: Residential care facilities.

### Data Analysis

Statistical analysis was performed using IBM SPSS Statistics 26. The exploratory factor analysis was performed using the principal components method and varimax rotation. Factor loadings (> 40) and *eigenvalues* over 1 were considered as criteria for retaining items in dimensions. The item was associated with each factor based on its factor loadings as well as the construct under analysis. In the case of items with factor loading below .40, each dimension’s underlying constructs were analyzed to choose the most adequate for each item. Reliability studies for the Portuguese version of the SAPDC were performed through internal consistency analysis. Cronbach’s α were calculated for the total scale and for each domain. The temporal stability (test-retest) was assessed by Intraclass Correlation Coefficient.

## Results

### Sample

Most respondents were direct-care workers (30.2%, *n* = 165). The majority of the sample, 70.3% (*n* = 384), had more than 12 years of schooling and the median time of work in gerontological care was 112 months (IQR = 120.50 months). The sample’s detailed data are described in Table 2.

**Table 2.** Sociodemographic and Professional Characteristics of the Participants (*N* = 546).

	*n*	*n* (%)/Mean (SD)/Median (IQR)
Age [range: 21–78]	525	40.43 (SD = 9.94)
Gender	542	
Female		518 (94.9%)
Male		24 (.7%)
Schooling	542	
Elementary school (from 1st to 4th grade)		3 (.5%)
Basic school (from 5th to 6th grade)		13 (2.4%)
Middle school (7th, 8th and 9th grades)		51 (9.3%)
High school (10th, 11th and 12th grades)		91 (16.7%)
Technological specialization courses		25 (4.6%)
Bachelors		4 (.7%)
Licentiate		267 (48.9%)
Masters		85 (15.6%)
Doctorate		3 (.5%)
Months of work in the area of older people care [range: 16–576]	529	Mdn = 112 IQR = 120.50
Professions	546	
Direct-care worker		165 (30.2%)
Technical director		158 (28.9%)
Social worker		26 (4.8%)
Nurse		54 (9.9%)
Psychologist		27 (4.9%)
Entertainment coordinator		61 (11.2%)
Others		55 (10%)
Type of organization management	540	
Private institution of social solidarity		449 (82.2%)
Private		91 (16.7%)
Number of users of the organization [range: 8–200]	528	Mdn = 44 IQR = 38
Geographical area	526	
Alentejo		39 (7.1%)
Algarve		13 (2.4%)
Madeira		3 (.5%)
Azores		10 (1.8%)
Lisbon area		27 (4.9%)
Center area		273 (50.0%)
Northern area		161 (29.5%)

### Psychometric Study

The descriptive characteristics for each item are in Table 3. Barlett test (*p* < .001) and the Kaiser-Meyer-Olkin’s sample adequacy measure (KMO = .954) indicated, according to Pereira and Patrício (2013), that performing the exploratory factor analysis was adequate to help in identifying the items’ underlying structure. Factor loadings and *eigenvalues* were considered as criteria for retaining items in dimensions. In the case of items 1, 4 and 36 (factor loading below .40), each dimension’s underlying constructs were analyzed to choose the most adequate for each item. These procedures resulted in the retention of 8 conceptually distinct dimensions, which the research and expert team considered coherent (Table 3).

**Table 3.** Description of Items, Factor Structure, Scale Content, Factor Loadings, Total Variance Explained and Cronbach’s α for the Portuguese Version of the Staff Assessment Person-Directed Care (*N* = 546).

Item			Subscales							
	Mean	SD	1	2	3	4	5	6	7	8
1	1.40	.87						**.330**		
2	1.68	1.05						**.584**		
3	2.38	1.28						**.612**		
4	3.39	1.20						**.311**		
5	2.07	1.17						**.669**		
6	2.30	1.12						**.765**		
7	1.89	1.14						**.630**		
8	3.20	1.28	**.660**							
9	2.98	1.28	**.639**							
10	3.34	1.23	**.779**							
11	3.47	1.18	**.776**							
12	3.58	1.22	**.744**							
13	3.76	1.16	**.738**							
14	3.50	1.25	**.683**							
15	3.42	1.17		**.716**						
16	3.02	1.27		**.700**						
17	3.65	1.11		**.693**						
18	3.88	1.09		**.702**						
19	3.44	1.21		**.758**						
20	3.55	1.19		**.761**						
21	2.63	1.25		**.642**						
22	3.50	1.26			**.607**					
23	3.66	1.25			**.655**					
24	3.12	1.42			**.673**					
25	3.32	1.36			**.705**					
26	3.27	1.34			**.630**					
27	3.43	1.39			**.699**					
28	3.96	1.10			**.640**					
29	3.81	1.23			**.623**					
30	4.22	1.04					**.774**			
31	2.59	1.43					**.582**			
32	4.07	1.14					**.775**			
33	2.34	1.41					**.451**			
34	3.49	1.32					**.677**			
35	3.55	1.31					**.484**			
36	3.75	1.33								**.325**
37	3.03	1.42								**.484**
38	3.34	1.29								**.600**
39	2.85	1.52								.**691**
40	3.42	1.28				**.448**				
41	4.43	.89				**.400**				
42	3.88	1.31							**.709**	
43	3.59	1.45							**.718**	
44	3.59	1.46							**.714**	
45	3.65	1.22				**.656**				
46	3.88	1.28				**.788**				
47	3.95	1.27				**.813**				
48	4.22	1.06				**.779**				
49	4.01	1.18				**.625**				
50	3.33	1.52				**.632**				
Scale and subescales’ means (standard deviations)	165.74 (36.78)		23.83 (7.07)	23.59 (6.76)	28.06 (7.80)	30.87 (7.31)	20.25 (5.75)	15.09 (5.23)	11.05 (3.70)	12.97 (4.13)
Cronbach’s α	.961		.918	.915	.886	.864	.840	.785	.860	.728
Explained variance (%)	62.2		35.6	5.7	4.6	4.2	3.7	3.4	2.7	2.3
Test-retest reliability coefficient	.951		.808	.853	.926	.926	.843	.743	.911	.795

Without referring to the themes defined by the SAPDC’s authors, the factors obtained in the study were analyzed and a theme was assigned. The first factor (items 8–14) had items related to personhood. The items in the second factor (items 15–21) focus on knowing the person. The third factor (items 22–29) assesses the comfort care construct. The fourth factor (40, 41, 45–50) shows the perception of organizational management and care culture. The fifth factor (items 30–35) represents the theme of social support networks. The sixth factor is composed of items (1–7) related to autonomy and decision-making. The seventh factor deals with cooperative and interdisciplinary teamwork (items 42–44). Lastly, the eighth factor (36–39) gathers items focused on personalizing the organizational context and tailoring the environment for residents.

The Cronbach’s α internal consistency coefficient (Table 3) was .961 for the whole scale. Cronbach’s α would not rise in any relevant way by excluding any item. Therefore, the Portuguese version of the SAPDC kept the originally proposed composition of 50 items. This factorial structure explains 62.2% of the sample’s total variance. The test-retest reliability coefficient was .951. for the total scale. Table 3 shows the psychometric data in detail.

## Discussion

Person-centered care is an alternative to traditional care and can foster a paradigm change that maximizes quality, dignified and sustainable care. Even though in the last two decades person-centered care has progressed significantly on an international level (Sköldunger et al., 2020), related research and formal application in Portugal is still rare. The scientific advancement of person-centered care requires measurement tools, and this study aims precisely at adapting and validating the SAPDC for the Portuguese population. Concerning the adaptation process, all relevant procedures related to the instrument’s cultural adjustments were performed. This guaranteed technical and scientific, linguistic, semantic, idiomatic, experiential and contextual equivalences between the original SAPDC and the Portuguese version. Validation procedures occurred through the psychometric study of the instrument’s adapted version.

Even though the original instrument’s authors performed two different sets of data analysis for the two major constructs identified in theoretical research (central dimensions of person-centered care and physical/organizational environment), the present study applied an exploratory factor analysis as a way to help identify the underlying structure of the 50 items. This procedure revealed an eight-factor solution. When comparing the results obtained with the original instrument’s sample in detail, the number of factors and item composition corresponded, with the exception of items 40 (“Do you have the information you need to support new client/resident choices?“) and 41 (“Are you able to be an advocate for residents/clients?“). Of the eight themes assigned to each dimension, six corresponded with residual linguistic adaptations to those presented by the original instrument’s authors (“Autonomy, Personhood, Knowing the Person, Comfort, Supporting relationships, Personal Environment for Residents”; White et al., 2008, p. 121). The exceptions were “management structure” and “your work with residents” (White et al., 2008, p. 121) which had no equivalent in our team’s assigned themes. This can be explained by the different retention of items 40 and 41. It is considered that this study’s retention and organization of items is adequate and, when compared to the original instrument, it also presents greater cohesion and content alignment of items in factors 4 (“perception of organizational management and care culture”) and 7 (“cooperative and interdisciplinary team work”).

Reliability studies were performed through internal consistency analysis and the total scale showed a very good internal consistency (α = .96) according to criteria by Pereira and Patrício (2013). This result is in line with the study of Martínez et al. (2016) for the Spanish population (α = .98). As for the internal consistency of subscales in relation to the same criteria, subscales 1 and 2 showed very good consistency (α > .90), and the remaining subscales (3–8) showed good internal consistency (α > .70). Similar results were reported in the original sample (α ranging between .74 and .91; White et al., 2008), and in a study performed with Canadian long-term care homes (Hunter et al., 2015).

Concerning temporal stability, according to criteria presented by Koo and Li (2016), the value obtained in the total scale (> .90) is considered to have excellent reliability. Using the same authors’ criteria as reference, subscale 6 is the only one presenting moderate value (< .75). The remaining subscales show good reliability (1, 2, 5 and 8) and excellent reliability (3, 4 and 7). The results show that the Portuguese version of the SAPDC has an adequate temporal stability, just like the Spanish population study by Martínez et al. (2016).

In summary, the psychometric study results show that the Portuguese version of the SAPDC is valid and reliable in the context for which it was adapted. This new validity evidence is added to those of other studies performed with different populations (e.g., Hunter et al., 2015; Martínez et al., 2016; Sullivan et al., 2012) and show that the SAPDC is a relevant tool for the study of person-centered care.

### Strengths, Limitations and Future Research

A limitation of this study is the inability to calculate the answer rate due to the inexistence of official data on the exact number of workers at RCFs and the use of the snowball method to distribute the answer protocol. Although all RCFs included in the official contact list were contacted, given the study’s confidentiality, it is not known which facilities divulged the study and it’s not possible to know how many staff members replied at each RCF.

The process of translating, adapting, back translating and validating the Portuguese version of the SAPDC was complex and time-consuming. To ensure the highest possible methodological rigor, combining directives and carefully planning the procedures was crucial. Another relevant strength is the characteristics of human resources participating in this study, namely the highly qualified experts and translators that facilitated the increase of methodological soundness. The opportunities given to participants of the pilot application to provide improvement suggestions was also a key aspect, as it provided validation of adequate terminology. Although the use of the snowball method resulted in the aforementioned limitation, it became helpful in obtaining a sample with significant dimension that was critical to the psychometric study. Besides, answers were obtained from professionals working at institutions in all regions of mainland Portugal and the islands. The larger amount of answers was obtained from the north and center, which is proportional to the larger concentration of RCFs in those areas (Ministry of Labor, Solidarity and Social Security, 2020).

Applying the SAPDC is fundamental to access the perception of staff on the level of person-centered care practiced at RCFs. The exercise of answering the SAPDC has in itself an awareness effect, as the staff must reflect on care practices before answering. The individual analysis of subscales and items may help identify improvement factors, which is especially useful in developing interventions. Within the context of evolving care culture, new research may include longitudinal studies that describe changes throughout time.

A combined strategy in terms of information sources is advantageous to avoid partial assessments of care. Therefore, future studies must include other sources and the voice of those living at RCFs, which would provide their perspective on the care received, and an understanding on how RCFs can adapt to their current users.

## Conclusion

The Portuguese version of the SAPDC showed adequate psychometric properties. Its application is therefore considered valid, reliable and adequate for measuring person-centered care in the context of the Portuguese RCFs through self-reporting from staff. This tool is expected to have practical implications for professional and research purposes and to be useful to identify improvement factors, support informed decisions, define policies, as well as to guide work practices and directives. Since the SAPDC is one of the most used instruments internationally, the existence of a Portuguese version may promote cooperative bonds and the interchangeability of data. This study aims to contribute to the existence of valid and reliable tools to assess person-centered care at Portuguese RCFs, a step that may maximize the advance of care approaches by increasing their respective quality.
